# Stem Cells in Cancer Progression and Therapy

**DOI:** 10.1155/2019/3507604

**Published:** 2019-03-05

**Authors:** Hua Tan, Weijun Su, Xing-Ding Zhang

**Affiliations:** ^1^School of Biomedical Informatics, The University of Texas Health Science Center at Houston, Houston, TX 77030, USA; ^2^School of Medicine, Nankai University, Tianjin 300071, China; ^3^School of Medicine, Sun Yat-sen University, Guangzhou, Guangdong 510080, China

Stem cells have been widely studied in the fields of cancer biology. Yet, many fundamental questions regarding the specific roles of stem cell in cancer progression and therapy remain elusive, impeding the effective clinical translation of stem cell research. On the one hand, cancer stem cells (CSCs), which represent a small population within tumors, with potential to differentiate into heterogeneous tumor cell colonies, are suggested to be responsible for therapy resistance and tumor recurrence. On the other hand, stem cells can be used to treat hematological malignancies through bone marrow transplantation by replenishing hematopoietic stem cells (HSCs). Stem cells play different roles in particular contexts.

Since first discovered in acute myeloid leukemia (AML) by CD34^+^/CD38^−^ biomarkers in 1997, CSCs attracted intensive efforts of research. Following the first evidence of CSCs existing in human cortical glial tumors, CSCs were identified in diverse human tumor tissues, including the breast, colon, and pancreas. Basically, CSCs display distinct biophysiological properties and responses to various treatments, compared to terminally differentiated cancer (TDC) cells. Therapeutic paradigm targeting on CSCs rather than bulk tumor cells (i.e., the TDCs) proved to be more potent in shrinking tumors.

The continuing advancement of molecular and cellular biology, especially the rapid progress in next-generation sequencing techniques and cancer genomics analysis approaches, is making therapeutics targeting CSCs more precise and effective. The single-cell sequencing technology and spatial transcriptomics become increasingly popular and powerful in profiling individual cells with genetic and epigenetic hierarchy. Versatile gene editing techniques such as CRISPR/Cas9 have also been harnessed to study CSCs in human tumors. Collectively, the molecular signatures of CSCs as well as CSC-initiated tumor cell lineage, such as gene mutation, gene and protein expression, microRNA regulations, DNA methylation, and histone modification, can be discerned and even edited in an unprecedentedly high resolution, which provides a unique opportunity for designing targeted and immune-based therapies.

Cancer has long been considered as a genetic disease, where genetic evolution of cancer cells shapes the tumor progression. Gene mutation is a stochastic process and hence results in unsynchronized mutation patterns in cells. Meanwhile, epigenetic markers changed dramatically over tumor evolution. Therefore, cancer cells in a tumor form a dynamic ecosystem of multiple colonies with heterogeneity in molecular signatures. Some of the colonies exemplify stem-like cell properties and are suggested to sustain the growth and expansion of the whole system. This complex and ever-changing system can be depicted and simulated by mathematical models. During the past years, mathematical models have played a critical role in generating and testing hypothesis regarding cancer progression. Tumor growth under various conditions including drug treatment has been well addressed by computational models. Vital biological processes, such as CSC lineage commitment, genetic evolution, oncogenic signaling cascades, neovascularization, and tumor cell-microenvironment interactions, have been carefully investigated. Systems biology approaches integrating experimental data, bioinformatics analysis, and computational model are expected to play an increasingly significant role in studying CSC-initiated and driven human cancer progression. The challenging part is to generate adequate yet nonredundant biomedical data at various scales (spatial and temporal) for model construction, calibration, and validation, towards enhancing the prediction capabilities of the in silico models.

While CSCs are deemed culprit of most therapy resistance and cancer relapse, stem cells derived from normal tissues can be leveraged to treat human malignancies. Via transplantation, HSCs were used to treat leukemia by replenishing the diminished blood cell population. Neural stem cells and human umbilical cord blood-derived mesenchymal stem cells (MSCs) were genetically transformed to treat gliomas. In these stem cell-based anticancer therapies, engineered stem cells stably express cancer suppressor genes or function as nanomedicine carriers to deliver cytotoxic agents targeting cancer cells. Strikingly, stem cells are also found to play an important role in cancer immunotherapy, by continuously replenishing the exhausted anticancer T-cells. Despite technique advancement, challenges existed. In particular, due to incomplete interpretation of the underlying molecular mechanisms, the treatment efficacy and durability of human cancers remain suboptimal in many cases. Additionally, the risk of tumorigenic transformation of the transplanted stem cells cannot be ignored. Upon being recruited into tumor microenvironment, stem cells might be educated into “cancer-favored” and reciprocally fuel tumor cells. These multifaceted interactions between stem cells and cancer cells should be taken into account while considering antitumor therapy with stem cells.

In the current special issue, we have solicited research articles and reviews that are aimed at addressing the biological and clinical questions regarding CSC-driven tumor progression and cancer treatment resistance and stem cell-based anticancer therapy. We stress on the molecular basis of how CSCs adapt to sustain the tumor progression under both physiological and stiff conditions such as local hypoxia and anticancer drug delivery. We also desire work exploring biological processes under stem cell transplantation therapy, such as how the stem cells home to the local environment, differentiate into progeny tumor cells, and/or exert paracrine effects.

B. Chen et al. explored the potential safety issues in the application of human bone marrow mesenchymal stem cells (hBM-MSCs) to regenerative medicine and tissue engineering. They revealed that hBM-MSC-conditioned medium (hBM-MSC-CM) promotes gastric cancer development via upregulation of c-Myc by both *in vitro* and *in vivo* experiments, which may be a potential risk factor and/or a therapeutic target for clinical applications.

D. Liu et al. conducted an integrative genome-wide analysis on gene expression and DNA copy number variations in a rare but aggressive malignancy—primary small-cell esophageal carcinoma (SCEC). They carried out a de novo expression array on three matched sets of primary SCEC and adjacent normal tissue samples procured from their institutional tissue bank. Stem cell-related pathways, WNT and Notch signaling, were shown to play significant roles in SCEC in this study.

M. Maldonado et al. studied the impact of human Wharton's jelly-derived MSCs (hWJ-MSCs) in *in vitro* maturation (IVM) of cumulus oocyte complexes (COCs). They demonstrated that hWJ-MSCs' differentiation potential and the presence of coordinated paracrine interaction between the stem cells and COCs are two prerequisites for the hWJ-MSCs to improve the IVM of COCs. Under appropriate conditions, the paracrine factors produced in the coculture system with DMEM-F12 may help develop synthetic media for desired *in vitro* culture of COCs. This work stands for a good attempt to research the paracrine effect imposed by stem cells on the cultured cells, especially using novel WJ-derived stem cells. The strategy and conclusions may have ramifications for cancer treatment via stem cell transplantation.

Y. Shi et al. addressed the differentiation of human umbilical cord mesenchymal stem cells (hUC-MSCs) into neuron-like cells. Working on human umbilical cord tissue, they proved that edaravone, a low-molecular antioxidative agent, can dose-dependently induce hUC-MSCs to differentiate into neuron-like cells. This study provides a novel method for neural-lineage induction from MSCs and demonstrates the potential applications of MSCs in regenerative medicine.

Y. Jiao et al. investigated the mechanisms underlying hUC-MSC-based cancer therapy. They evaluated the effect of secreted factors of hUC-MSCs on the breast cancer cell line MCF7 in terms of morphological changes, cell viability, cell cycle, apoptosis, DNA fragmentation, and interleukin-1*β* (IL-1*β*) secretion and confirmed that the secreted factors of hUC-MSCs could cause MCF7 cell death by inducing pyroptosis. Their results may enable the community to better understand the effect of hUC-MSCs on cell-based breast cancer therapy, as well as its associated molecular basis.

While this special issue represents an exciting start point to address the fundamental questions regarding stem cells in cancer biology and therapy, many challenges regarding the clinical effectiveness and biosafety still remain to be conquered, as mentioned above. We appeal more intense attention and participation in this promising field in the future, especially the involvement of new concepts and technologies such as single-cell sequencing, spatial transcriptomics, gene editing, and immunotherapy in the stem cell-related cancer research. We expect a new era in which stem cells can be elaborately tuned to fight for cancers, with detrimental effects trimmed and merits maximized.

